# The complete mitochondrial genome of *Scatoglyphus polytrematus* (Acari: Acaridae)

**DOI:** 10.1080/23802359.2021.1927874

**Published:** 2021-05-19

**Authors:** Yu Fang, Li Tao, Xiaoqian Zhou, Luyao Liu, Feiyan Li, Qianqian Yang, Xingquan Xia, Shulin Zhou, Entao Sun

**Affiliations:** a Department of Health Inspection and Quarantine, Wannan Medical College, Wuhu, China; b Department of Medical Parasitology, Wannan Medical College, Wuhu, China; c School of Medicine & Holistic Integrative Medicine, Nanjing University of Chinese Medicine, Nanjing, China; d College of Life Science, The Provincial Key Lab of the Conservation and Exploitation Research of Biological Resources in Anhui, Anhui Normal University, Wuhu, China

**Keywords:** Astigmatina, Acaridae, mitochondrial genome, *Scatoglyphus polytrematus*

## Abstract

We assembled and annotated the complete mitochondrial genome of *Scatoglyphus polytrematus*. It is the first complete mitochondrial genome sequence from the genus *Scatoglyphus*. The mitogenome was 13,966 bp in length and contains 37 genes (including 13 protein-coding genes, 22 transfer RNA (tRNA), and two ribosomal RNA (rRNA)), and one largest non-coding region. The gene arrangement of *S. polytrematus* is consistent with the pattern of possible common ancestor of astigmatid mites. In the present study, phylogenetic analysis shows that genus *Scatoglyphus* was clustered into one branch with other Acaridae species.

Mites of the family Acaridae are economically important polyphagous pest commonly living on stored products and also responsible for allergic reactions to humans (Cui 2014). *Scatoglyphus polytrematus* (Berlese 1913) belongs to Astigmatina, Acaridae, which was the only species reported in genus *Scatoglyphus*. To date, six mitogenomes from species of Acaridae have been sequenced. Three mitochondrial transfer RNA (tRNA) genes (*trnF*, *trnS1*, and *trnQ*) were reported as lost in *Tyrophagus longior* (Yang and Li 2015). Here, we present the complete mitogenome of *S. polytrematus*, analyze its composition.

We collected samples of *S. polytrematus* from piles of firewood in Wuhu, southeast China (118°38′E, 31°33′N), in July 2019. Voucher specimen was deposited in the herbarium of Department of Health Inspection and Quarantine, Wannan Medical College (Entao Sun, asdentao@126.com) (under the accession number WNMC0820190410). Mites were stored in 100% ethanol at −20 °C until use. The whole-genomic DNA was extracted by standard phenol–chloroform extraction (Zhang and Alvarado 2018). Sequencing libraries were prepared by Shanghai BIOZERON Company (Shanghai, China) and sequenced on the Illumina Hiseq 4000 (San Diego, CA). The assembled genome was annotated using the MITOS WebServer (Bernt et al. 2013). The PCGs boundaries were confirmed manually by MEGA X software (Kumar et al. 2018), and BLASTp (Altschul et al. 1997). We annotated tRNAs using ARWEN (Laslett and Canback 2008), tRNAscan-SE (Schattner 2005), and manual identification based on the anticodon and predicted secondary structure.

**Figure 1.**
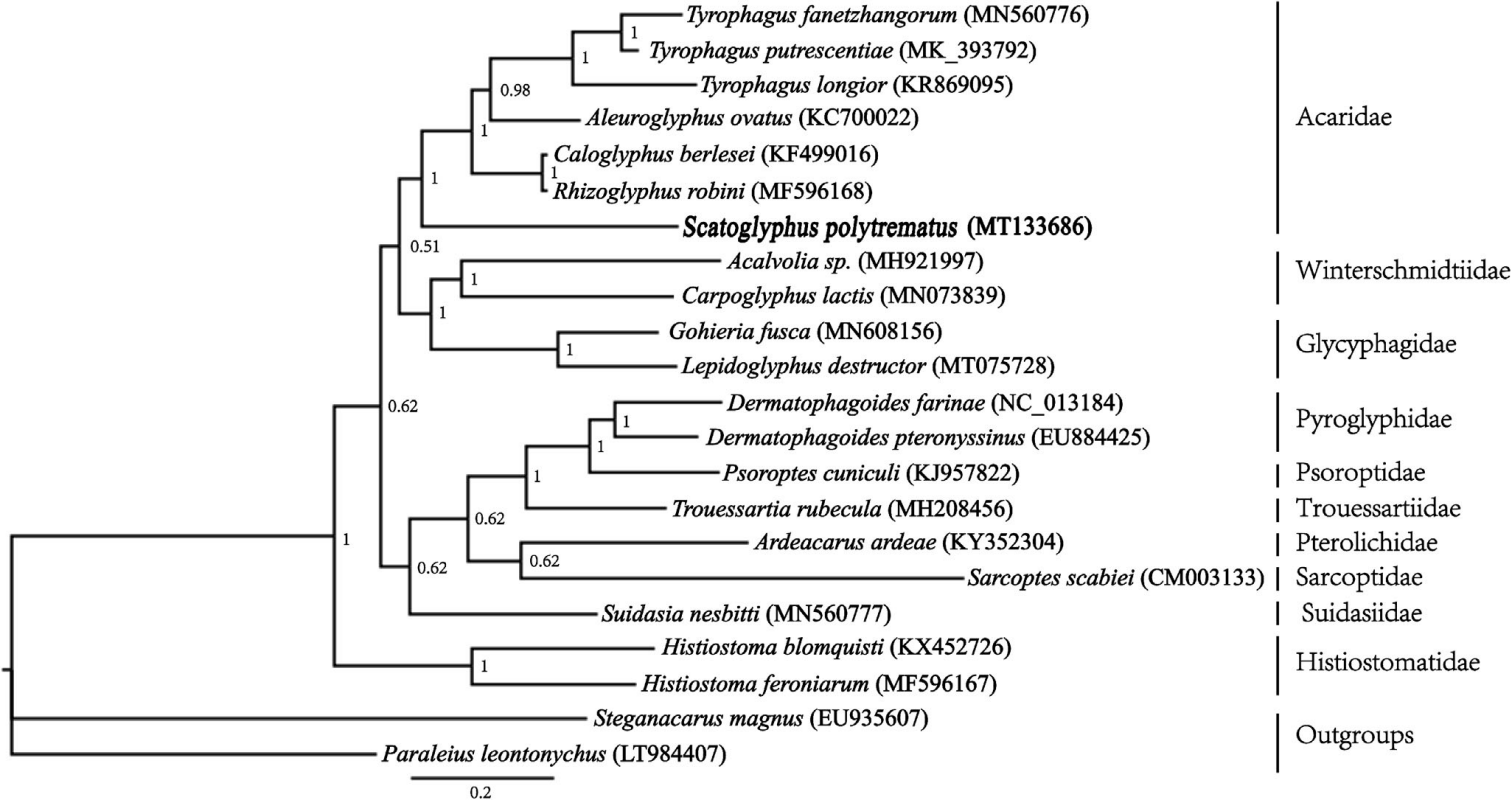
Phylogenetic tree inferred from mitochondrial genome sequences using Bayesian methods. Branch lengths presented here follow the Bayesian methods analysis. Node numbers indicate Bayesian posterior probabilities (BPPs).

The complete mitogenome of *S. polytrematus* (GenBank: MT133686) is 13,966 bp. The overall base composition of the entire *S. polytrematus* mitogenome consisted of 24.1% A, 46.1% T, 10.2% C, and 19.6% G, resulting in a negative AT-skew (-0.3125) and a positive GC-skew (0.3122). The genome contains 37 genes, including 13 protein-coding genes (PCGs), 22 tRNA genes, two ribosomal RNA (rRNA) genes, and one D-loop. The gene arrangement of *S. polytrematus* is consistent with the most available astigmatid mites, which is supposed to reflect the possible common ancestor of astigmatid mites (Li and Xue 2019). The length of the tRNAs ranged from 47 to 62 bp. Only the *trnK* showed the typical cloverleaf. Other tRNAs showed the reduction of tRNA-D- and/or T-arms, like those found in other astigmatid mites.

To infer the phylogenetic position of *S. polytrematus* within the Astigmatina, we generated a data set of 22 mite taxa (20 astigmatid mites and two oribatid mites), including the nucleotide sequences and amino acid sequences of the 13 PCGs. The nucleotide and amino acid sequences of the PCGs were aligned separately using the TranslatorX server (Abascal 2010), where MAFFT is used to build the protein alignment (Katoh and Standley 2013). For the nucleotide sequences, translation was done under the invertebrate mitochondrial genetic code. The large gaps and ambiguous sites were deleted by Gblocks v.0.91b (Castresana 2000). Phylogeneticanalyses were conducted using Bayesian inference (BI) (Ronquist et al. 2012) method. The phylogenetic analysis supported the monophyly of Acaridae (Figure 1). The genus *Scatoglyphus* was placed under family Acaroidae, which is congruent with the current classification systems (Krantz and Walter 2009).

The complete mitogenome of *S. polytrematus* was determined in this study. This information from our study has important ramifications for understanding of mitogenome evolution in astigmatid mites.

## Data Availability

Mitogenome data supporting this study are openly available in GenBank at: https://www.ncbi.nlm.nih.gov/nuccore/MT133686. Associated BioProject and BioSample accession numbers are https://www.ncbi.nlm.nih.gov/bioproject/608033 and SAMN14116537, respectively.
